# Trend of incompleteness of maternal schooling and race/skin color variables held on the Brazilian Live Birth Information System, 2012-2020

**DOI:** 10.1590/S2237-96222023000100013

**Published:** 2023-05-08

**Authors:** Bárbara Estevam Ferreira Santana, Amanda Cristina de Souza Andrade, Ana Paula Muraro

**Affiliations:** 1 Universidade Federal de Mato Grosso, Programa de Pós-Graduação em Saúde Coletiva, Cuiabá, MT, Brazil

**Keywords:** Information Systems, Birth Certificates, Maternal Educational Status, Racial Groups, Time Series Studies, Brazil., Sistemas de Información, Actas de Nacimiento, Nivel Educativo Materno, Grupos Raciales, Estudios de Series Temporales, Brasil, Sistemas de Informação, Declaração de Nascido Vivo, Escolaridade Materna, Grupos Raciais, Estudos de Séries Temporais, Brasil

## Abstract

**Objective:**

to analyze the trend of incompleteness of the maternal schooling and race/skin color variables held on the Brazilian Live Birth Information System (SINASC) between 2012 and 2020.

**Methods:**

this was an ecological time series study of the incompleteness of maternal schooling and race/skin color data for Brazil, its regions and Federative Units, by means of joinpoint regression and calculation of annual percentage change (APC) and average annual percentage change.

**Results:**

a total of 26,112,301 births were registered in Brazil in the period; incompleteness of maternal schooling data decreased for Brazil (APC = -8.1%) and the Southeast (APC = -19.5%) and Midwest (APC = -17.6%) regions; as for race/skin color, there was a downward trend for Brazil (APC = -8.2%) and all regions, except the Northeast region, while nine Federative Units and the Federal District showed a stationary trend.

**Conclusion:**

there was an improvement in filling out these variables on the SINASC, but with regional disparities, mainly for race/skin color.

## Resumo

**Objetivo:**

analisar a tendência da incompletude das variáveis escolaridade e raça/cor da pele da mãe no Sistema de Informações sobre Nascidos Vivos (Sinasc), Brasil, entre 2012 e 2020.

**Métodos:**

estudo ecológico de série temporal sobre a incompletude da escolaridade e da raça/cor da pele da mãe para o Brasil, suas macrorregiões e Unidades da Federação, pela regressão por *joinpoint*, e cálculo da variação percentual anual (VPA) e da variação percentual anual média.

**Resultados:**

foram registrados 26.112.301 nascimentos no Brasil, no período; no país (VPA = -8,1%) e em suas regiões Sudeste (VPA = -19,5%) e Centro-Oeste (VPA = -17,6%), houve redução da incompletude da escolaridade materna; quanto à raça/cor da pele da mãe, observou-se queda para o Brasil (VPA = -8,2%) e todas as suas regiões, exceto o Nordeste, e nove UFs e o Distrito Federal com tendência estável.

**Conclusão:**

o preenchimento das variáveis no Sinasc melhorou, porém com disparidades regionais, principalmente quanto à raça/cor da pele.

## Resumen

**Objetivo:**

analizar la tendencia de incompletitud de educación y raza/color de piel materna en el Sistema de Información de Nacidos Vivo (Sinasc), Brasil, entre 2012-2020.

**Métodos:**

estudio ecológico de serie temporal sobre la incompletitud de la educación y raza/color de piel materna para Brasil, regiones y Unidades de la Federación (UF), a través de regresión de joinpoint y cálculo de cambio porcentual anual (APC) y cambio porcentual anual promedio

**Resultados:**

se registraron 26.112.301 nacimientos en Brasil en el período. Brasil (APC = -8,1%) y regiones Sudeste (APC = -19,5%) y Centro-Oeste (APC = -17,6%) disminuirán la incompletud de la educación materna. En cuanto a raza/color de piel, hubo un descenso para Brasil (APC = -8,2%) y todas las regiones, excepto Nordeste, y nueve UF y Distrito Federal presentaron tendencia estacionaria.

**Conclusión:**

e llenado das variables en el Sinasc ha mejorado, pero con disparidades regionales, principalmente por raza/color de piel.

**Table:** 

Study contributions	
**Main results**	A decrease was found in the incompleteness of maternal schooling and race/skin color data between 2012 and 2020 for Brazil as a whole, although with regional disparities, especially regarding maternal race/skin color.
**Implications for services**	We identified Brazilian regions (Northeast and South) and Federative Units where actions are necessary to further improve SINASC information and contribute with strategies to address health inequalities.
**Perspectives**	Good quality SINASC data is important for monitoring and critical analysis capable of informing the planning and evaluation of health policies, actions and services in Brazil and its Federative Units.

## INTRODUCTION

The Brazilian National Health System (Sistema Único de Saúde - SUS) has equity as one of its doctrinal principles, that is, the recognition of social differences and diversity in each individual’s health condition and health needs. To this end, it is essential that Health Information System data collection documents contain variables that are sensitive to this aspect, enabling indicators to be built and information to be produced that can be used to reduce social and health inequalities,^1^ whereby people’s race/skin color and schooling stand out as markers of socioeconomic status.

The Brazilian Health Information Systems have been designed to support health management and care. Standing out among the main systems is the Live Birth Information System (Sistema de Informações sobre Nascidos Vivos - SINASC), intended to hold birth records throughout the national territory.^1^ Production of incorrect data can generate erroneous interpretations and evaluations of situations under assessment, and can lead to erroneous interventions and planning that may not contribute to the improvement of the population’s health.^2^ ^,^ ^3^

The importance of the SINASC is related to its providing data for calculating essential indicators, such as infant and maternal mortality rates, birth rates, vaccination coverage, in addition to assisting in health diagnoses, surveillance and monitoring of newborns, identifying availability and use of childbirth services, as well as supporting the formulation of maternal and child health policies.^4^

Evaluation of data quality has been carried out based on certain indicators, such as coverage, completeness, reliability and consistency. Although Szwarcwald et al.^5^ found that in 2011 and 2012, SINASC coverage of information on live births was high and homogeneous, with coverage of more than 90% of live births in most of the country’s Federative Units, other studies at the national level, carried out with data up to 2013, indicated differences in the completeness of information between Brazil’s five macro-regions and highlighted maternal schooling and race/skin color as being among the most incomplete variables/fields.^6^ ^-^ ^8^ A recent literature review of studies published from 2010 to 2018, also pointed to race/skin color as being among the variables with higher frequencies of incompleteness.^9^

This fact is of special importance, since the sociodemographic variables held on the SINASC allow identification of social and health inequalities in several health outcomes relating to women and children, such as access to health services, prenatal and childbirth care and maternal-infant mortality. Information on maternal schooling, race/skin color and occupation, low birth weight, and type of child delivery are sensitive indicators for assessing health inequities. Therefore, analysis of the level of completeness of this information in recent years is necessary for evaluating and monitoring the complex data collection process.^10^

Another important factor in this area is the identification of regional disparities. Nationwide studies, such as those by Romero & Cunha^11^ and Silvestrin et al.,^8^ conducted in 2007 and 2018, respectively, point out differences between the country’s macro-regions with regard to the incompleteness of variables. Therefore, this investigation is considered important in order to identify these interstate and regional differences. 

The objective of this study was to analyze the trend of incompleteness of the maternal schooling and race/skin color variables for births reported on the SINASC in Brazil between 2012 and 2020.

## METHODS

This was an ecological time series study of annual records of live births held on the Brazilian SINASC for the period 2012-2020. The data were retrieved in May 2021 from the SUS Department of Information Technology (DATASUS) website - https://datasus.saude.gov.br/transferencia-de-arquivos/ -, by means of a manual review, using files available in DBC format. Subsequently, the data were compressed using the executable version of the Tab for Windows (TabWin) program in DBF format and adapted to a CSV file, executable by Microsoft Excel 2010. The database was built using DATASUS information on the maternal schooling and race/skin color variables, for each year, for Brazil as a whole and for its Federative Units, including the Federal District.

In 2011 changes were made to the Live Birth Certificate (Declaração de Nascido Vivo - DNV), including changes to the maternal schooling and race/skin color variables. Since then, maternal schooling refers to the last grade completed, and should be answered in two steps: firstly, the “Level” of education attended (1 - Elementary; 2 - Junior High; 3 - High School; 4 - Incomplete Higher Education; 5 - Complete Higher Education); and second, the “Grade” completed, if levels 1, 2 or 3 are informed. If the mother has no level of education, the field “No education” should be filled out. “Unknown”^9^ is also an option available when filling out this field. The information on maternal race/skin color is self-reported by the mother and should be filled out with one of the following sub-variables: 1. White; 2. Black; 3. Asian; 4. Mixed race; 5. Indigenous. This sub-variable does not allow the “Unknown” option and we therefore considered fields left blank in order to assess incompleteness.^12^

We calculated percentage incompleteness (blank or unknown) for the maternal schooling race/ skin color variables, for each geographic region of the country (North, Northeast, South, Midwest, and Southeast) and Federative Unit, year by year. The degree of incompleteness was classified according to the criteria defined by Romero & Cunha:^11^ excellent, when the variable has less than 5% incompleteness; good (from 5.0% to 9.9% incompleteness); regular (from 10.0% to 19.9%); poor (from 20.0% to 49.9%); and very poor (50.0% incompleteness or more). 

We prepared thematic maps by regions of the country and Federative Units for the years 2012, 2016 and 2020, showing percentage incompleteness of the maternal schooling and race/skin color variables. The map class intervals were defined based on the criteria proposed by Romero & Cunha.^11^ The maps were prepared using the open source TabWin program, based on the geographic grid provided by the Brazilian Institute of Geography and Statistics (Instituto Brasileiro de Geografia e Estatística - IBGE) and accessed by DATASUS. 

We used joinpoint regression to analyze the trend of incompleteness of the maternal schooling and race/skin color variables, taking the year as the regressor variable (2012 to 2020), with stratification by region of the country and Federative Units, using Joinpoint Trend Analysis, version 4.9.1. The joinpoint regression model is a way of analyzing temporal trends, evaluating joinpoints and whether there are changes in the pattern of this trend. We performed logarithmic transformation of percentage incompleteness to reduce regression analysis residual variance heterogeneity. Our assumptions as to serial autocorrelation, normality, and homoscedasticity of the residuals were verified by the Durbin-Watson, Shapiro Wilk, and Breusch-Pagan tests, respectively, using Stata version 16. We used a 5% significance level.

 We calculated: (i) Annual Percentage Change (APC) for each segment; and (ii) Average Annual Percentage Change (AAPC), which is the weighted geometric average of the different APCs, with weighting equal to the segment size for each time interval.^13^ ^,^ ^14^ The selection of the number of joinpoints to obtain the significant model was done using the software’s default settings. When the trend was not significant, it was considered to be stationary, that is, it did not show a statistically significant increase or decrease in its time series. When the incompleteness trend increased, this indicated worsening in the filling out of variables; while a decreasing trend indicated improvement. 

This research was conducted in accordance with the ethical precepts in force and necessary for conducting research with human beings, as determined by National Health Council Resolution No. 466, dated December 12, 2012, and also by the Access to Information Law No. 12,527, dated November 18, 2011. This study used open access data available in the information systems and therefore did not require submission to a Research Ethics Committee. 

## RESULTS

**Figure 1:**
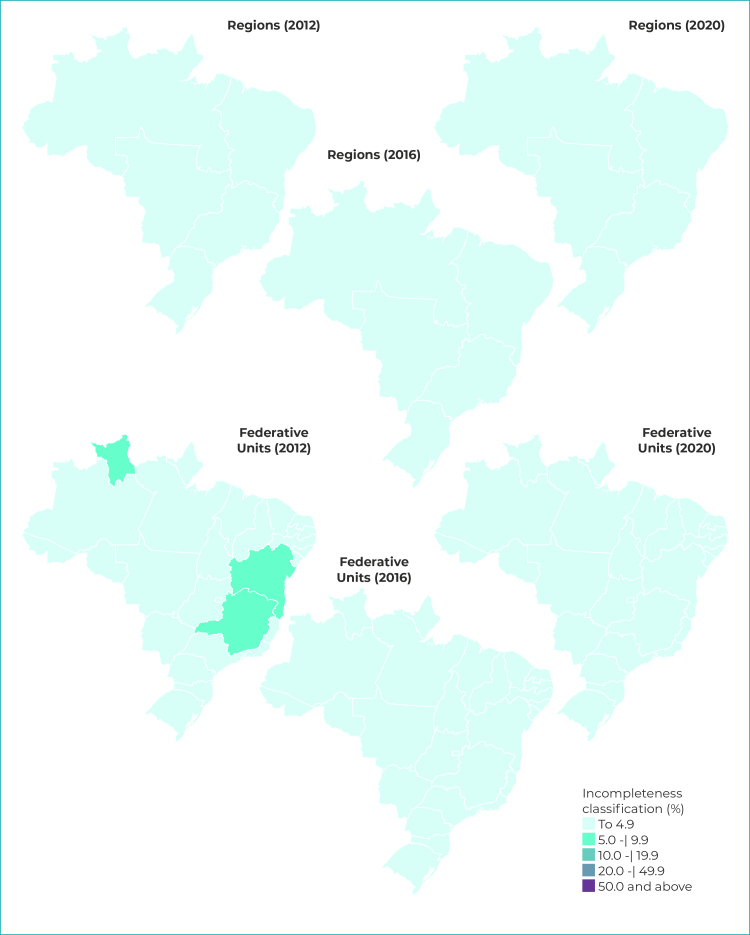
- Classification^a^ of percentage incompleteness of maternal schooling by geographic region and Federative Unit, Brazil, 2012, 2016 and 2020

a) Classification as per Romero & Cunha.^11^

A total of 26,112,301 births were recorded in Brazil between 2012 e 2020, with 2,901,367 births per year on average.

### Maternal schooling

**Figure 2:**
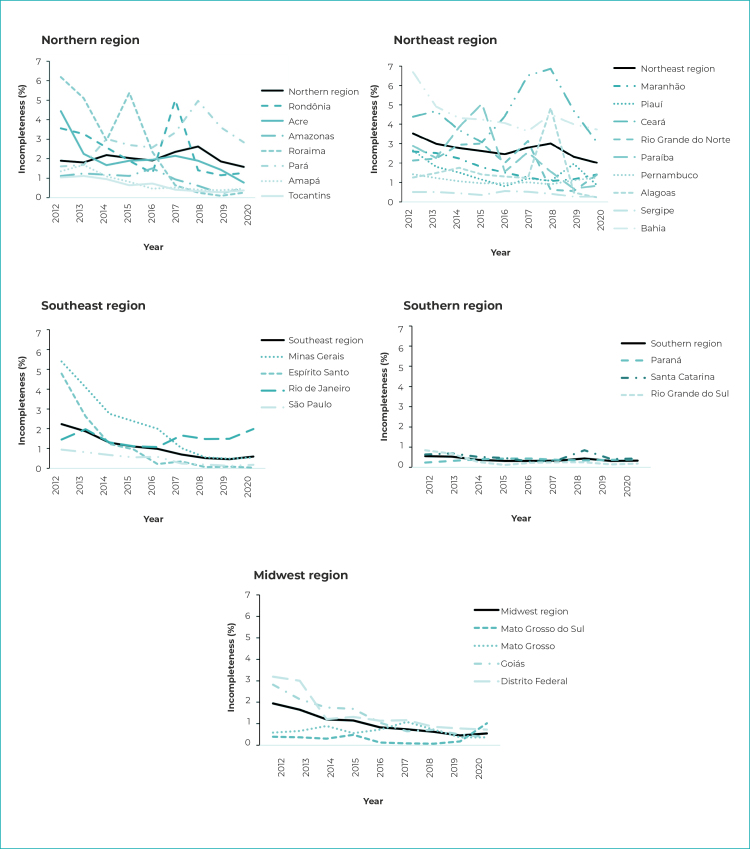
- Percentage incompleteness of maternal schooling per year, by geographic region and Federative Unit, Brazil, 2012-2020

In 2012, only Roraima, Bahia and Minas Gerais had incompleteness between 5% and 9.9% for maternal schooling; the remaining Federative Units had excellent classification (up to 4.9% incompleteness). In 2016, all geographic regions of the country and all Federative Units had excellent completion, a fact that continued in the last year analyzed, 2020 (Figure 1). The Northeast and Northern regions showed greater variability of incompleteness between their Federative Units, while throughout the period incompleteness for the Southern region as a whole remained close to that of its Federative Units and was less than 1% (Figure 2).

Regarding temporal trends, maternal schooling incompleteness decreased about 8% per year, taking Brazil as a whole; when evaluating the country’s regions, however, only the Southeast and the Midwest showed a downward trend in the completeness of this variable, with APC of -19.5% and -17.6%, respectively (Table 1).

**Table 1:** - Annual percentage change and average annual percentage change of birth records on the Live Birth Information System, in relation to maternal schooling and race/skin color, by geographic region and Federative Unit, Brazil, 2012-2020

	Maternal schooling				Maternal race/skin color			
	Period^a^	APC^b^ (95%CI)^d^	Trend	AAPC^c^ (95%CI)^d^	Period	APC^b^ (95%CI)^d^	Trend	AAPC^c^ (95%CI)^d^
Brazil	2012-2020	-8.1 (-10.9;-5.3)	Falling	-8.1 (-10.9;-5.3)	2012-2020	-8.2 (-12.5;-3.6)	Falling	-8.2 (-12.5;-3.6)
**Northern region**	2012-2020	2.3 (-2.7;7.6)	Stable	2.3 (-2.7;7.6)	2012-2020	-8.2 (-14.8;-1.0)	Falling	-8.2 (-14.8;-1.0)
Rondônia	2012-2020	2.2 (-13.5;20.7)	Stable	2.2 (-13.5;20.7)	2012-2020	2.5 (-14.3;22.6)	Stable	2.5 (-14.3;22.6)
Acre	2012-2020	-13.6 (-21.5;-4.9)	Falling	-13.6 (-21.5;-4.9)	2012-2014 2014-2020	35.4 (-5.1;93.4) -30.8 (-40.2;-19.9)	Stable Falling	-18.1 (-25.9;-9.6)
Amazonas	2012-2016 2016-2020	6.5 (-2.1;15.8) -32.0 (-44.0;-17.5)	Stable Falling	-14.9 (-21.0;-8.3)	2012-2020	-4.4 (-21.4;16.1)	Stable	-4.4 (-21.4;16.1)
Roraima	2012-2020	-13.6 (-26.9;2.0)	Stable	-13.6 (-26.9;2.0)	2012-2020	-5.6 (-18.9;10.0)	Stable	-5.6 (-18.9;10.0)
Pará	2012-2020	11.0 (1.2;21.7)	Rising	11.0 (1.2;21.7)	2012-2020	-7.1 (-15.6;2.2)	Stable	-7.1 (-15.6;2.2)
Amapá	2012-2020	-21.4 (-26.9;-15.4)	Falling	-21.4 (-26.9;-15.4)	2012-2020	-1.6 (-14.1;12.7)	Stable	-1.6 (-14.1;12.7)
Tocantins	2012-2020	-16.5 (-21.2;-11.5)	Falling	-16.5 (-21.2;-11.5)	2012-2020	-11.2 (-17.3;-4.5)	Falling	-11.2 (-17.3;-4.5)
**Northeast region**	2012-2020	-3.4 (-8.0;1.6)	Stable	-3.4 (-8.0;1.6)	2012-2020	-0.4 (-3.3;2.7)	Stable	-0.4 (-3.3;2.7)
Maranhão	2012-2020	-11.8 (-15.7;-7.8)	Falling	-11.8 (-15.7;-7.8)	2012-2020	-9.1 (-19.0;2.1)	Stable	-9.1 (-19.0;2.1)
Piauí	2012-2016 2016-2020	-22.9 (-30.4;-14.5) 21.4 (5.4;39.7)	Falling Rising	-3.3 (-9.0;2.9)	2012-2015 2015-2020	-20.4 (-32.6;-6.0) 9.4 (1.3;18.1)	Falling Rising	-2.9 (-8.2;2.6)
Ceará	2012-2020	6.9 (-7.1;23.0)	Stable	6.9 (-7.1;23.0)	2012-2017 2017-2020	11.6 (1.1;23.2) -7.1 (-26.0;16.6)	Rising Stable	4.2 (-3.3;12.2)
Rio Grande do Norte	2012-2020	-3.3 (-13.8;8.4)	Stable	-3.3 (-13.8;8.4)	2012-2016 2016-2020	22.7 (-1.5;53.0) -53.6 (-79.6;2.6)	Stable Stable	-24.5 (-43.6;0.9)
Paraíba	2012-2015 2015-2020	23.7 (-18.7;88.3) -32.1 (-54.6;1.5)	Stable Stable	-15.0 (-31.1;4.8)	2012-2020	3.5 (-2.8;10.2)	Stable	3.5 (-2.8;10.2)
Pernambuco	2012-2015 2015-2020	-13.3 (-25.1;0.3) 4.3 (-1.9;10.9)	Stable Stable	-2.7 (-7.2;2.0)	2012-2020	4.0 (-1.2;9.4)	Stable	4.0 (-1.2;9.4)
Alagoas	2012-2020	10.0 (-9.7;34.1)	Stable	10.0 (-9.7;34.1)	2012-2020	25.2 (12.8;39.1)	Rising	25.2 (12.8;39.1)
Sergipe	2012-2020	-2.4 (-9.6;5.4)	Stable	-2.4 (-9.6;5.4)	2012-2016 2016-2020	24.9 (16.0;34.5) -37.0 (-44.7;-28.4)	Rising Falling	-11.3 (-15.9;-6.5)
Bahia	2012-2014 2014-2020	-19.5 (-40.9;9.5) -1.0 (-5.0;3.2)	Stable Stable	-6.0 (-11.3;-0.3)	2012-2020	-5.8 (-8.5;-3.0)	Falling	-5.8 (-8.5;-3.0)
**Southeast region**	2012-2020	-19.5 (-22.2;-16.8)	Falling	-19.5 (-22.2;-16.8)	2012-2020	-21.4 (-28.0;-14.1)	Falling	-21.4 (-28.0;-14.1)
Minas Gerais	2012-2020	-24.2 (-29.0;-19.0)	Falling	-24.2 (-29.0;-19.0)	2012-2020	-14.6 (-21.7;-6.8)	Falling	-14.6 (-21.7;-6.8)
Espírito Santo	2012-2020	-45.0 (-46.6;-43.3)	Falling	-45.0 (-46.6;-43.3)	2012-2020	-28.1 (-30.7;-25.4)	Falling	-28.1 (-30.7;-25.4)
Rio de Janeiro	2012-2015 2015-2020	-16.9 (-43.2;21.8) 10.2 (-2.1;24.2)	Stable Stable	-0.8 (-11.5;11.1)	2012-2020	-3.5 (-12.0;5.8)	Stable	-3.5 (-12.0;5.8)
São Paulo	2012-2020	-17.7 (-23.5;-11.4)	Falling	-17.7 (-23.5;-11.4)	2012-2014 2014-2020	-58.8 (-71.9;-39.8) -16.9 (-25.7;-7.0)	Falling Falling	-30.3 (-36.2;-23.7)
**Southern region**	2012-2015 2015-2020	-17.7 (-36.0;5.9) 1.8 (-9.5;14.5)	Stable	-6.0 (-13.6;2.3)	2012-2014 2014-2020	-30.3 (-39.4;-19.7) -10.1 (-13.4;-6.6)	Falling Falling	-15.6 (-18.3;-12.9)
Paraná	2012-2020	0.3 (-5.7;6.6)	Stable	5.7 (-5.0;17.7)	2012-2020	0.2 (-3.7;4.1)	Stable	0.2 (-3.7;4.1)
Santa Catarina	2012-2020	0.1 (-10.4;11.8)	Stable	0.1 (-10.4;11.8)	2012-2020	-14.5 (-16.5;-12.4)	Falling	-14.5 (-16.5;-12.4)
Rio Grande do Sul	2012-2020	-19.3 (-28.5;-9.0)	Falling	-19.3 (-28.5;-9.0)	2012-2014 2014-2020	-63.4 (-74.0;-48.5) -18.4 (-25.2;-11.0)	Falling Falling	-33.2 (-38.1;-27.9)
**Midwest region**	2012-2020	-17.6 (-18.8;-16.5)	Falling	-17.6 (-18.8;-16.5)	2012-2016 2016-2020	9.4 (2.6;16.6) -16.4 (-23.4;-8.7)	Rising Falling	-4.4 (-8.0;-0.6)
Mato Grosso do Sul	2012-2020	10.2 (-8.8;33.1)	Stable	7.7 (-27.3;59.5)	2012-2020	-26.2 ( -40.4;-8.7)	Falling	-26.2 ( -40.4;-8.7)
Mato Grosso	2012-2020	2.1 (-9.0;14.5)	Stable	2.1 (-9.0;14.5)	2012-2016 2016-2020	21.1 (8.6;35.1) -16,7 (-24,8; -7,7)	Rising Falling	0.4 (-4.7;5.9)
Goiás	2012-2020	-21.0 (-24.8;-16.9)	Falling	-21.0 (-24.8;-16.9)	2012-2016 2016-2020	11.6 (7.5;15.9) -31.1 (-35.8;-26.0)	Rising Falling	-12.3 (-14.8;-9.8)
Distrito Federal	2012-2014 2014-2020	-37.9 (-49.3;-23.8) -11.7 (-14.5;-8.7)	Falling Falling	-19.1 (-22.3;-15.8)	2012-2018 2018-2020	8.9 (2.9;15.3) -30.9 (-65.7;39.0)	Rising Stable	-2.8 (-14.4;10.4)

a) In the joinpoint time series analysis, when there is a change in the trend pattern, the periods are separated and the final year of one period coincides with the initial year of the following period; b) APC: Annual percentage change; c) AAPC: Average annual percentage change calculated by logarithmic regression, according to joinpoints at each period of change; d) 95%CI: 95% confidence interval.

In the Northern region, the states (Federative Units) of Acre, Pará, Amapá and Tocantins had significant percentage change. Except for the state of Pará, where an 11% increase in the change in incompleteness was found, the remaining Northern region Federative Units showed a downward trend. In the state of Amazonas, there was a change in the trend in 2016, going from stationary to falling (Table 1).

In the Northeast region, the incompleteness trend was stationary, with the exception of the state of Maranhão (APC = -11.8%) and the state of Piauí, which had a downward trend followed by a rising trend between 2016 and 2020 (Table 1). The Southeast region showed a downward trend for incompleteness (APC = -19.5%), with only the state of Rio de Janeiro having a stationary trend, furthermore this state had low incompleteness (around 2%) right from the beginning of the period. These results are similar to those of the Midwest region, with a drop of 17.6% per year in the incompleteness of maternal schooling, with only the state of Mato Grosso and Mato Grosso do Sul being stable (Table 1).

### Maternal race/skin color

**Figure 3:**
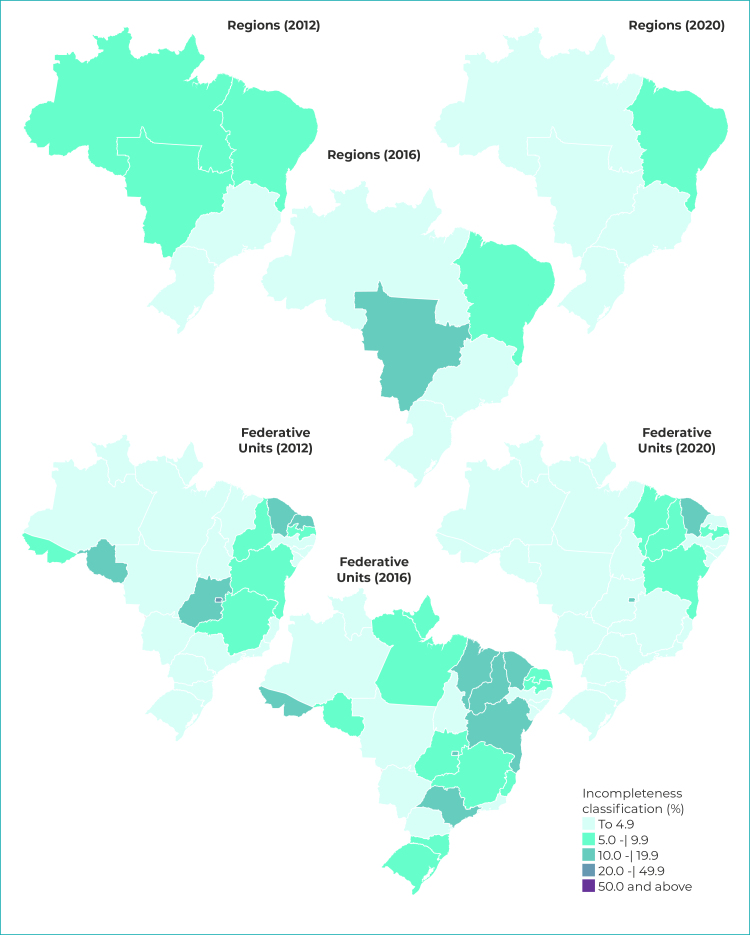
- Classification^a^ of percentage incompleteness of maternal race/skin color by geographic region and Federative Unit, Brazil, 2012, 2016 and 2020

**Figure 4:**
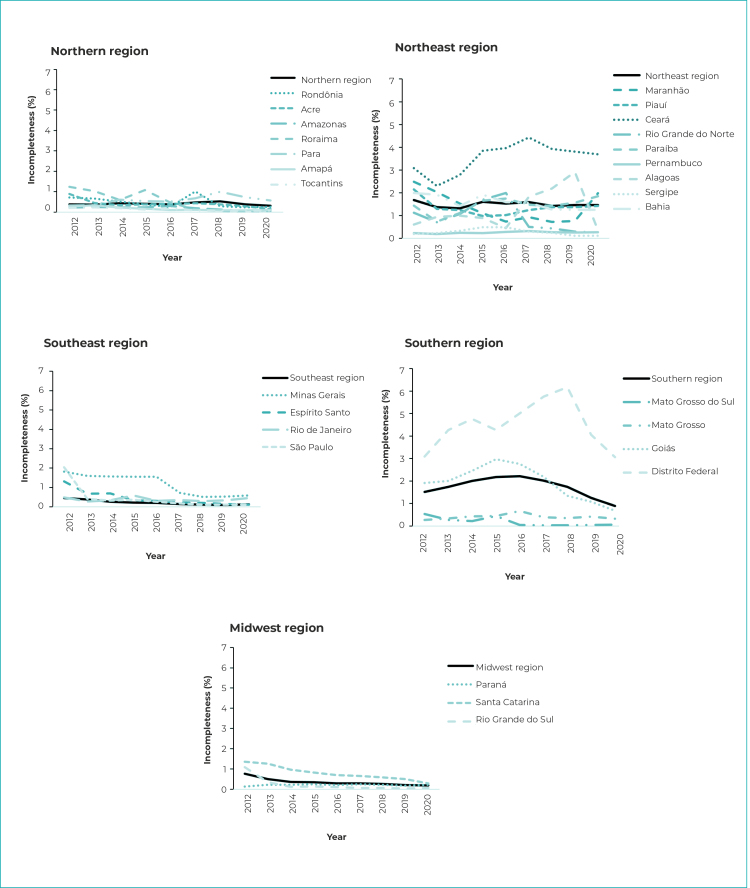
- Percentage incompleteness of maternal race/skin color schooling per year, by geographic region and Federative Unit, Brazil

The degree of incompleteness of the maternal race/skin color variable in 2012 was classified as regular (between 5.0% and 9.9%) in Federative Units of the North, Northeast and Midwest regions. In 2016, incompleteness in the Midwest region was classified as poor (between 10.0% and 19.9%) and in 2020, only the Federative Units of the Northeast region had regular classification (Figure 3). During the period analyzed, the state of Ceará, in the Northeast region, and the Federal District, in the Midwest region, stood out with the highest rates of incompleteness (Figure 4).

a) Classification as per Romero & Cunha.^11^

Regarding the incompleteness trend (Table 1) for Brazil as a whole, a significant decrease was found (APC = -8.2%). All regions, except the Northeast, had a downward trend in race/skin color incompleteness. The Southeast and Northern regions also had a falling trend, with -21.4% APC and -8.2% APC, respectively. In the Southern region, the first segment of the trend happened between 2012 and 2014, with -30.3% annual change. In the Midwest region, a change in the trend was found: the first period (2012-2016) was characterized by a rising trend (APC = 9.4%), followed by a falling trend (APC = -16.4%). 

In the Northeast the results were varied, notably with increased incompleteness found in the state of Alagoas. Ceará had an increase followed by stability, while and Piauí and Sergipe had a falling trend followed by rising trend in incompleteness (Table 1). In the Southeast region, Rio de Janeiro was the only state that did not have a falling trend, while São Paulo was the only state for which there was a change in the trend. With respect to the Southern region, only the state of Paraná showed no significant trend, while Rio Grande do Sul stood out with a joinpoint in 2014 (Table 1). In the Midwest region, in all states and the Federal District there was a decrease in the incompleteness of the information on maternal race/skin color, except for the Federal District in the period from 2018 to 2020. In the states of Mato Grosso and Goiás, the characteristics were similar, with an upward trend between 2012 and 2016, followed by a decrease in incompleteness in subsequent years (Table 1).

### DISCUSSION

The findings of this study indicated improvement in the completeness of the maternal schooling variable on the SINASC between 2012 and 2020 for Brazil as a whole: right from the beginning of the period analyzed, none of the Federative Units had percentage of incompleteness higher than 10% for this variable. This is the first nationwide study to assess the trend of incompleteness of the maternal race/skin color variable, after its inclusion in the DNV: six Federative Units were classified as having regular completeness of this information in 2012, and there was a downward trend in the incompleteness of this variable for Brazil as a whole and for almost all its macro-regions, except the Northeast region, for which completeness of this variable was stable. 

The periods that showed a change in trend were diverse among the Federative Units, both for maternal schooling and for maternal race/skin color, notably those that indicated worsening incompleteness, such as Piauí. The incompleteness trend of the maternal schooling variable should be analyzed in light of the excellent completeness found since the beginning of the period (2012), when only Bahia, Roraima and Minas Gerais had incompleteness above 5%. This result corroborates that reported by Oliveira et al.,^6^ who found that completeness of maternal schooling was above 98% between 2006 and 2010 for Brazil as a whole. 

Silvestrin et al.^8^ focused on Brazilian state capitals and found a trend towards a reduction in incompleteness of the maternal schooling variable in the Southern region of Brazil during the period they analyzed (1996-2013), in all capitals of that region. This result is consistent with that found in the present study, in which all Southern region states showed an excellent degree of completeness with a stable trend right from the beginning of the period evaluated. 

In relation to maternal race/skin color, the Northeast region stood out as the only one without a significant trend of decreasing incompleteness. Moreover, the states of Ceará, Sergipe and Alagoas showed an increase in incompleteness of this variable in the period selected for this study. These results reinforce those found by Silva et al.^15^ also for the Northeast region, in an earlier period (2000- 2009), when the highest proportions of incompleteness corresponded to the variables on babies’ race/skin color and their clinical assessment using the Apgar Score, with no decrease in incompleteness of the information on race/skin color, contrary to what was found for most baby-related variables. 

It should be noted that in the present study, we evaluated the trend in the nine years following the last change to the SINASC data collection tool, i.e. the DNV, which underwent changes in 2011.^16^ The possible impact of this period of adaptation to the new forms of data collection and completion on the incompleteness results found should not be ruled out. There were changes directly related to the variables evaluated by this study, since with effect from 2011 maternal schooling is filled out according to education levels and the maternal race/skin variable was included, rather than that of the baby, whose race/skin color is reported by the mother.² Therefore, comparisons are hampered, since most studies address the period before the change, analyzing the trend of incompleteness in relation to the baby’s race/skin color,^11^ ^,^ ^15^ ^,^ ^16^ apart from studies that took both the baby’s and the mother’s race/skin color into consideration.^3^

The Ministry of Health’s National Policy on Health Information and Informatics (Política Nacional de Informação e Informática em Saúde - PNIIS) highlights that incompleteness is the result of a number of issues, such as

absence of information in medical records and women’s companions not knowing certain information, blank variables (not filled out) are a reflection of the lack of care and importance given to filling out the information by the health professional in charge.^10^ ^,^ ^16^

In addition, maternal sociodemographic and economic factors should be considered. A study analyzing birth records in low- and middle-income countries showed that maternal sociodemographic factors were also associated with lower completeness of maternal information, such as younger age group, higher number of previous births, lower educational level and socioeconomic status.^17^ These factors may be related to the greater incompleteness of records in Federative Units with a higher proportion of poverty and less educated inhabitants, such as the Federative Units in the Northeast region. 

A variety of factors are capable of contributing to low data completeness, such as illegible handwriting, poor flow of information within the health service, parturient women or their families unable to answer questions. SINASC user manuals lack clearness with regard to instructions on filling out fields, as well as greater interest in some variables to the detriment of others, are also cited as causes of incompleteness. In addition, incompleteness can be attributed to lack of attention and carelessness in filling out the DNV by the health professionals in charge.^15^ ^,^ ^18^ In view of this, it is essential to highlight the importance of training these professionals, emphasizing the relevance of the information, evaluating possible problems encountered and their solution, such as, for example, the revision of the DNV which took place in 2011 and the adoption of a new version of the form, with improvements, such as its shorter fields.

Previous studies have shown greater incompleteness in the initial years of the analysis, confirming the conclusions of this study, namely: in the first years, the Federative Units showed improvement.^4^ ^,^ ^8^ ^,^ ^11^ ^,^ ^16^ ^,^ ^19^ ^,^ ^20^ It should be noted that the analysis periods and regions are restricted in previous studies. Due to this, and due to the reality of regional disparities regarding Health Information System data filling out and quality, this investigation needs to be considered for other local levels, as well as paying attention to changes made over time.^15^ ^,^ ^20^

According to the National Policy on Health Information and Informatics, analysis of sociodemographic characteristics, such as race/skin color, schooling, age and gender, can support actions aimed at reducing health inequalities, in addition to encouraging the scaling up of service quality and humanization.^10^ Among the strategies to improve the completeness of information in vital statistics systems, Romaguera et al.^21^ suggest strategies such as active tracing of events in the routine of municipal health services, progress in death surveillance, integration of maternal, fetal and child death committees, systematic and periodic training and qualification of professionals involved in the production of information. 

In this study we used the cutoff points proposed by Romero and Cunha,^11^ adopted in most studies that have assessed the completeness of information from vital statistics systems in Brazil, including those that have specifically considered information on maternal schooling race/skin color, among the variables analyzed.^4^ ^,^ ^15^ ^,^ ^18^

The present study was limited to assessing maternal schooling and race/skin color data held on the SINASC, as this is useful information for assessing social inequalities in health; in addition to having already been identified as the least complete sociodemographic variables by other studies.^6^ ^-^ ^8^ However, it is recognized that there are other important variables, also presented in the literature, with low completeness, such as duration of pregnancy and clinical analysis using the Apgar Score,^16^ ^,^ ^22^ in addition to other quality dimensions essential for enabling evaluation of data accuracy. However, the results of our analysis evaluate the current system information, complementing studies carried out in previous years and serving as a basis for others, in Brazil’s regions and Federative Units. 

A limitation of this study is the statistical approach adopted. Joinpoint regression makes it possible to identify the magnitude, direction and points of change in the trends of incompleteness of the maternal schooling and race/skin color variables held on the SINASC. However, a disadvantage of using this method of analysis is the uncertainty in estimating the number of points of change, which may not correspond to the actual change.^23^

Given the above, it should be noted that health information can contribute and impact the practical reality of health actions and services, when available in an accurate manner, becoming a tool to address outcomes that are often preventable, as is the case of infant and maternal mortality. The results show discrepancies between the Brazilian macro-regions and even within the same region, regarding the incompleteness of two pieces of information that are widely used in the analysis of social determinants related to maternal and child health, namely, maternal schooling and maternal race/skin color. However, other types of analysis and the incorporation of information in planning practices and in the performance of health services are necessary, in order to increasingly enhance the information held on health information systems and thus contribute to improving the health of the population.

In the analyses by the country’s regions, we found discrepancies between the results of the Federative Units and this therefore demonstrates the importance of continuing studies that evaluate municipal health services or groupings, such as health regions, overcoming the limitation resulting from a more generalized evaluation and focusing on gaps and potentialities of specific territories. Finally, it is worth mentioning, as pointed out in the literature and in the present study, that the results are not homogeneous between Federative Units and regions, and the quality of the data depends on the specificities of the territory, such as conditions of human and technological development. There are intraregional differences that can be looked into in more depth, as well as intrastate studies, to assess the need for actions aimed at improving the completeness of these data.
